# On Idiotic Crania, Idiocy, and Cretinism
*Report on Idiotic Crania, Idiocy, and Cretinism. By Samuel Kneeland, Jun., M.D., Boston. Read before the Boston Society for Medical Improvement, Jan. 13, 1851. (American Journal of Science.)


**Published:** 1851-07-01

**Authors:** 


### ART. V.—ON IDIOTIC CRANIA, IDIOCY, AND CRETINISM.*

BEFORE examining minutely the subject of idiocy, it is difficult to understand the extreme differences and inconsistencies of authors who have made it a special study, and the almost impossibility of arriving at any conclusions on the subject. The divisions and subdivisions of the various forms of idiocy are innumerable; varying according to each author's appreciation of the mental, moral, and physical phenomena presented. An enumeration, even, of the different systems, would only confuse and perplex. A simple division may therefore be adopted, which, if inadmissible as a strictly scientific and accurate system, will suggest the most important considerations connected with idiocy.

Without going into particulars, we notice at once a great division of idiots into idiots from birth, and idiots from various causes subsequent to birth. In the first, or *congenital* idiocy, there is an arrest of cerebral development in the fœtus, indicated by a too small brain and by the external development and form of the anterior portions of the cranium; in the second, or acquired, *secondary* idiocy, the diminution or abolition of the mental powers depends on disorganization or disease in a previously normal and healthy brain, and may or may not be accompanied with change in the size and form of the cranium; this disease may be hydrocephalus, scrofulous hypertrophy, exhaustion from venereal excesses, hard study, &c., and various mechanical injuries. To the first class, we restrict the name of *idiots*, or what are expressed in the phrase "natural fools." To the second class, wherever idiocy is secondary, may be given, for want of a better, the name "*cretin*"—a term erroneously confined by many to a few miserable creatures in the Alpine and other mountain valleys. "Among their number may be ranked," in the words of Dr. Buckminster Brown, "the numerous individuals who are to be found scattered over every country, and who, under various names, such as innocents, simpletons, or idiots, are to be met with in the valleys of Vermont, New Hampshire, or Scotland, as well as Switzerland." Among the great class of cretins, we perceive also a natural division, according to the nature of the predisposing causes, into two orders: 1st. *Cretins*, or "fools," properly so called, in whom, while the brain is healthy at birth, there is some hereditary disease, as scrofula, syphilis, or other cause of weakness, which predisposes to idiocy during childhood, and which is frequently followed

* Report on Idiotic Crania, Idiocy, and Cretinism. By Samuel Kneeland, Jun., M.D., Boston. Read before the Boston Society for Medical Improvement, Jan. 13, 1851. (American Journal of Science.)

by it when external circumstances favour its development; this would include "cretins" from endemic causes in the valleys of mountainous districts, from hydrocephalus, or other chronic cerebral affections. 2nd. Persons often seen in insane asylums, in whom the understanding is completely lost, without passing through insanity; whose minds were once strong, but have been destroyed by various excesses; to use an expression in Dr. Howe's report, they are "demented."

Dr. Brown gives the following distinction between idiocy and cretinism: "In the latter, it is disease in the framework, it is the external avenues which are closed; in the former (idiocy), it is almost unchangeable mental conformation. Or, more properly speaking, in the latter (cretinism) it is an altered condition of the nerves, sensitive and motor, and of their peripheral ramifications; in the former (idiocy), it is to the great nervous centre alone that the evil is to be traced." He thus makes the pathological condition the element of difference; while we consider rather the nature of the causes as constituting the difference. These causes, congenital or secondary, may, we think, produce idiocy or cretinism, without reference to the pathological seat; in other words, that, though in idiocy the evil is seated in the great nervous centre (the brain), in cretinism this same nervous centre may be equally affected. It would seem, also, that the distinction by the causes offers better indications for prognosis and treatment than any difference in the pathological condition; as in the congenital affection, whether seated in the brain or in the nerves and framework, the prognosis would be unfavourable, and treatment probably useless; while in the secondary affection, where the cause is more generally appreciable, treatment will be more likely to be of advantage.

It is not easy to say much on the subject of idiocy without entering the domain of phrenology. However much we may affect to ridicule the idea that small heads are absolute indications of inferior intellect, we cannot deny a connexion between the *relative* size of certain parts of the brain and the *degree* of manifestation of the intellectual, moral, and animal sentiments. In idiots, the forehead is unusually low, and the intellect proportionably dull, corresponding to the arrest of development of the *anterior* cerebral lobes. In idiocy, there is no one peculiar form to the exclusion of others; there is every variety of intellectual and moral inferiority, which phrenology traces to a corresponding deficiency of brain. At any rate, Gall, Combe, and Spurzheim, have added a great deal to our knowledge of mental affections, their causes, symptoms, pathology, and treatment.

There can be no doubt that deficient cerebral development is a cause of idiocy, independent of any actual disease. Numerous examples are given by authors of full-grown idiots with brains no larger than those of infants, with no other mark of disease about them. The predisposing cause must act during gestation—idiocy may be hereditary; children of besotted parents are very apt to be idiotic. Of this it is needless to quote examples, as the records of idiocy are full of striking proofs of this visiting on the children the sin of their parents. Various shocks to the nervous system of the mother have been known to cause idiocy: thus, Esquirol mentions that, during the exciting period of the French Revolution, many women brought forth idiotic children, who before and after that period had healthy ones. The intermarriage of near relatives is very apt to be followed by idiotic children. In 359 cases alluded to by Dr. S. G. Howe, in his State Report, 17 were *known* to be the children of parents nearly related by blood, and doubtless many more should be added. This makes (so far as such few cases go) the proportion of idiots from this cause one-eighteenth of the whole; and, considering the small ratio such marriages bear to the great mass of marriages, this proportion becomes of more importance. In this report, it is said: "Most of the parents were intemperate or scrofulous; some were both the one and the other; of course there were other causes to increase chances of infirm offspring, besides that of the intermarriage. There were born unto them 95 children, of whom 44 were idiotic, 12 others were scrofulous and puny, one was deaf, and one was a dwarf. In some cases, all the children were either idiotic or very scrofulous and puny. In one family of eight children, five were idiotic."

Idiocy (as has just been defined), being congenital, cannot be said to have any *exciting* causes, or rather these are the same as the *predisposing*. In all the forms of cretinism, in addition to the predisposing causes of idiocy acting on the mother during gestation, there are various exciting causes; as, endemic influences of mountain valleys, improper lactation, accidents of dentition, convulsions, grave diseases of infancy and childhood, falls on the head, &c., acting on a brain prone to derangement from hereditary causes of weakness. In after life, a vicious system of education, a life of excess, may cause "dementia" in a perfectly developed brain. It has been said that continued compression of the child's head during labour may cause idiocy; and that any subsequent improper compression may have the same effect. This may be true as regards infants; but there are many facts against the latter. We know, for instance, that the artificial compression exercised by many nations (as the ancient Peruvians, the Natchez Indians, &c.), though distorting the cranium to a great degree, does not cause idiocy. The Peruvian forehead is as flat as the idiot's; but this *imitation* of nature is not followed by the natural consequence, idiocy.

According to the best authority, the number of idiots and cretins in Massachusetts must be at least 1200, in a population of one million, or about one in every 830 individuals.

There is no one peculiar form of idiotism, or cretinism. There is every variety and gradation, from the most degraded brutish idiot to the imbecile with a feeble, yet perceptible, intellect. It would be useless and tedious to give here anything like a full account of the different *symptoms*. It will be sufficient to allude to the stupid physiognomy, inability or indisposition to move, deformity, dulness or abolition of the senses, inability to articulate, involuntary discharges, insufficient and sluggish circulation, in idiots proper; and in the various forms of cretinism (in many cases apparently synonymous with rachitis, scrofula, epilepsy, hydrocephalus), to the distorted features, convulsive movements, disgusting habits, depending on the activity of certain instincts unrestrained by moral or intellectual principles. In this class must be placed those whose idiocy depends on congenital absence of certain senses, which Broussais thought justified him in saying that persons born blind and deaf are necessarily idiots. But numerous exceptions show that the absence of these senses is not incompatible with considerable intellectual powers: the case of Laura Bridgman need only be mentioned to prove this.

Cretins and idiots usually have the animal and instinctive propensities active, even when there appears no spark of reason or human sentiment; but to these may be added various faculties, as memory, order, a disposition to destroy, secrete, or steal. Some have considerable mechanical talent, *e. g.*, the cretins employed at Geneva in making parts of watches; some are gentle and affectionate, others the opposite; some have the organic functions perfect, others not. Dr. Rush says, "I once saw a man who discovered no one mark of reason, and yet possessed the moral sense and faculty in so high a degree that he spent his whole life in acts of benevolence."

This will suffice for the symptoms proper of idiocy and cretinism; but a curious fact may be here introduced in regard to the diseases of this class of persons, viz., that they are accompanied with very little reaction, and are very difficult to diagnosticate from the absence of the classical symptoms: in inflammation of the lungs, for instance, the circulation and respiration are very little, if at all, accelerated; rusty sputa either do not exist, or they are swallowed as by children; all their diseases seem to take on the chronic and latent form; acute affections and cerebral inflammations rarely terminate their lives, and the gravest accidents and dangerous wounds are borne without any great constitutional disturbance.

It is the opinion of many authors that cretinism is one of the many forms in which the scrofulous diathesis shows itself; and certainly the external signs of the so-called "lymphatic temperament," the complexion, features, proportions, &c., are strongly in favour of this view. In idiots proper, the nature of their affection must be attributed rather to an arrest of development than to any constitutional disease, though upon true idiots may be found marks of scrofula. As any well-ascertained connexion between cretinism and other disease is important as regards treatment, the relation of rachitis to cretinism may be here mentioned. In the "Dictionary of Medical Sciences" of Berlin (according to Mr. Güggenbuhl's First Report, pp. 47–8) it is said that autopsies of cretins prove that their cranial bones have undergone a softening similar to that found in rachitis, and evident marks of this disease have been found in other parts of the skeleton; the symptoms from the beginning are similar, but not precisely alike, in cretinism and rachitis. Without admitting absolute identity, the authors think that the differences depend on this, "that cretinism, taking its departure from the cranium and brain, soon attacks the physical and intellectual powers; while rickets, commencing in the trunk and the extremities, may make great progress before exciting any grave trouble in the system." A careful examination made by the physicians of the Canton Vallais, in Switzerland, has shown (according to the above report) that at least one-half of the cases of cretinism there commence by the symptoms of rachitis, principally softening of the bones. According to Esquirol, and others since his time, cretinism is usually developed between the second and fifth years, and almost never after seven years of age.

The pathological anatomy of idiocy and cretinism presents very various conditions. In true congenital idiocy, we find a brain (healthy perhaps) too small for the full manifestation of the moral and intellectual faculties; there is an arrest of development of the anterior and middle portions, corresponding to the seats of these faculties as given by phrenologists.

This deficiency in the anterior region of the skull is well shown by the series of casts and crania exhibited in the following tables. Measurements have been carefully taken in three directions, which will be compared with normal heads, in order to sustain the exactness of Dr. Gall's law in reference to the dimensions of the skull necessary for the full exercise of the faculties.

TABLE I.—*Idiots.*

| Sex. | Age. | 1st Measurement. | 2nd Measurement. | 3rd Measurement. | Where found. | No. |
|---|---|---|---|---|---|---|
| Male .... | 25 | 15 inches. | 10 inches. | 9 inches. | No. 407, Mass. Med. Col. | 1 |
| Female .. | 17 | 15 " | 10 " | 10 " | No. 406 do. | 2 |
| " | 60 | 18 " | 11 " | 10 " | No. 89 do. | 3 |
| Male .... | Adult. | 18 " | 10 " | $10\frac{1}{2}$ " | No. 89 do. | 4 |
| " | " | 18 " | 11 " | 11 " | No. 90 do. | 5 |
| Female .. | " | 18 " | $10\frac{3}{4}$ " | $10\frac{1}{2}$ " | No. 91 do. | 6 |
| " | " | $18\frac{1}{4}$ " | 10 " | 11 " | No. 92 do. | 7 |
| " | " | $17\frac{1}{2}$ " | $10\frac{1}{2}$ " | 10 " | No. 93 do. | 8 |
| — | ? | 14 " | $8\frac{1}{2}$ " | $8\frac{3}{4}$ " | No. 94 do. | 9 |
| — | 6 | 14 " | $8\frac{1}{4}$ " | $8\frac{1}{4}$ " | No. 96[a] do. | 10 |
| — | 21 | $14\frac{1}{2}$ " | 9 " | 9 " | No. 96[b] do. | 11 |
| S. Walker | 6 | — | $10\frac{1}{8}$ " | $10\frac{1}{2}$ " | Dr. Howe's Report. | 12 |
| G. Rowell | 9 | $14\frac{9}{10}$ " | $10\frac{1}{8}$ " | $10\frac{1}{2}$ " | Ditto | 13 |
| E. S. Field | 7 | 17 " | $11\frac{3}{4}$ " | $11\frac{1}{10}$ " | Ditto | 14 |
| Charles .. | 16 | 16 " | 8 " | 9 " | Institution at S. Boston. | 15 |
| Rowell girl | 8 | $15\frac{1}{2}$ " | $10\frac{1}{4}$ " | $10\frac{1}{2}$ " | Ditto | 16 |
| Aztec boy | 7 | 12 " | $7\frac{1}{2}$ " | $7\frac{1}{2}$ " | Described by Dr. Warren. | 17 |
| Aztec girl | 5 | 12 " | $7\frac{1}{2}$ " | $7\frac{1}{2}$ " | | 18 |

The first measurement is the circumference of the cranium just above the superciliary ridges, passing through the most prominent part of the occiput; the second is from the root of the nose to the occipital protuberance, over the top of the head; the third is from one auditory meatus to the other, also over the top of the skull. Dr. Gall has laid down the rule, that when the first is less than seventeen inches, and the second less than eleven inches, or even twelve, there is always greater or less stupidity; that, when the first is eleven to thirteen inches, and the second eight or nine, the intellectual faculties cannot be exercised. With reference to this point, Andral, as quoted by Dr. Combe (on Insanity, p. 264), says: "As a general rule, it may be stated, also, that, when the circumference of the head is only between twelve and fifteen inches, the mental condition can be but little above idiocy. Eighteen inches may be regarded as the circumference necessary for intelligence; at twenty, the mental faculties are still more developed; and from twenty to twenty-two inches they attain their maximum power."

Eleven of the above eighteen idiots are doubtless adults; or, if not, it is of little consequence, as it will be seen in the next table that these measurements are less than the normal measurements of a child four years old. For the first (the circumference) the smallest is 14 inches (with the exception of the Aztec children), and the largest $18\frac{1}{4}$—all below the size necessary for much intelligence; the second varies from $8\frac{1}{4}$ to $11\frac{3}{4}$ inches; and the third from $8\frac{1}{4}$ to $11\frac{1}{4}$.

It may be well to glance here at some of the most prominent points exhibited by these casts and crania of idiots, which, to one conversant with phrenology, would suggest many very interesting questions. All show a preponderance of the animal propensities, some of one kind, some of another. Nos. 1 and 2 were noted for pride, self-esteem, and combativeness. No. 3, a woman sixty years old, who, during her youth, had the cerebellum active, had sufficient locality to wander from home and find her way back again; she was very fond of colours, and submitted to be cast on receiving a gaudy-coloured shawl. Nos. 4 to 8, inclusive, were members of one family, all of whom, seven in number, were idiots; their parents were frequently in a beastly state of intoxication. Nos. 5, 6, 7, and 8, so far as the relative size of the various regions of the skull was concerned, had phrenologically good heads, but their absolute size was too small for intelligence. Nos. 9 and 10 approximate very nearly to the orang-outang, as will be seen by reference to the second table. No. 10, the famous idiot of Suabia, six years old, by the flat forehead, vertex, and occiput, and prominent features, resembles much the so-called Aztec children, of whom we have given the measurements in Nos. 17 and 18. No. 11, the idiot of St. Denis, is of good shape, but of remarkable smallness, considering the subject was twenty-one years old. Nos. 13 and 16 are brother and sister, and are interesting, as showing one of the causes of idiocy, viz., intemperance in the parents. On the authority of Mr. Richards, we may state that the parents of these children have had healthy and normal offspring at periods of their lives when intemperance was not their prominent vice, and that, too, both before and since the birth of these, who were born while their parents were addicted to strong drink. No. 16 also has club-foot. No. 15, lately received at South Boston, is a most curious-looking idiot; sixteen years of age, of large frame and great strength, his broad shoulders surmounted by a small head, covered with bristly red hair; his lower extremities are weak, and his gait shuffling; the circulation is very languid, as it generally is in idiots, indicated by the lividity and coldness of the lips and hands; his violent gestures and uncouth noises have been considerably modified by kind treatment during his stay of only a few weeks. Of the Aztec children we shall speak below.

TABLE II.

| | 1st Measure. | 2nd Measure. | 3rd Measure. | Where found. | No. |
|---|---|---|---|---|---|
| Average skull .. | 21 inches | 12½ inches | 12½ inches | | 19 |
| Carib skull .... | 21¼ " | 12 " | 12 " | No.359, Medical College | 20 |
| Child 5 years old | 18½ " | 12 " | 12¼ " | Nat. Hist. Society | 21 |
| " 4 " | 18¾ " | 11½ " | 12½ " | Do. | 22 |
| " 3 " | 18 " | 11¼ " | 12 " | Do. | 23 |
| " 2 " | 17½ " | 12 " | 12¾ " | Do. | 24 |
| " 1 " | 17½ " | 11¼ " | 12¼ " | Do. | 25 |
| " at birth .. | 12¼ " | 8 " | 8 " | Do. | 26 |
| Orang Outang | 13½ " | 8 " | 8½ " | No. 97, Medical College | 27 |

From these tables, it will be perceived that all the idiots had skulls too small for a brain sufficient for a full manifestation of the intellectual powers; that they had skulls smaller than an average child of five years of age; that two, one adult and the other seven years old, had skulls inferior to a child two years old; that six were inferior to a child one year old in measurements, though the ages ranged from six to twenty-one years; that four were not above the new-born child as regards cerebral development; that a diminution of three inches in the first measurement, and one to two inches in the second and third measurements, is almost sure to be followed by greater or less imbecility. In the second table, by comparing the flattened Carib skull with an average skull, we see that, though the form is changed, and the forehead much flattened, the capacity of the cranium is unchanged; showing that distortion is no indication necessarily of imbecility, unless confirmed by actual measurement. There would, perhaps, be a difference of half an inch in different specimens of skulls of children; but a single average specimen is sufficient to show the inferiority of the idiot skull.

We see, then, that below a certain size of the brain, there is idiocy; and we also see that the actual size of idiotic brains varies. An idiot with a small brain (*e. g.* No. 13) may be superior to one with a larger brain (*e. g.* No. 14); in both, idiocy arises from a too small brain: but why should the smaller be the better brain? It is due to the different conditions of the bodily organization, or *temperament.* Dr. Howe, speaking of these two boys in his report, says: "The first-named boy, whose head is so much smaller than the second, and, indeed, than any boy in the school, and who has such a striking resemblance to the ape tribe, manifests much more vivacity, activity, and intelligence than the second, and, indeed, than several of the others —and precisely for the reason that the man of 'blood,' or fine temperament, is superior in these respects to the man of coarse organization— though his brain may be smaller. The boy's body is of a much finer organization, and his brain, doubtless, is so likewise."—p. 57.

The deformity of idiot crania affects principally the anterior and superior portions; while the parts destined for the animal propensities and instincts are well developed. This we should expect from an arrest of development; as it is admitted that, when the growth of the brain is natural, the anterior portions are hardly formed at a time when the convolutions of the other regions are comparatively well developed —showing the order and preference of nature in forming first the portions destined for the vegetative functions.

The fact that in idiots the animal, instinctive, and emotional sensaions are usually active, is interesting in connexion with certain views regarding the physiology of the brain that have of late years been generally received. At the base of the brain, distinct from the cerebral hemispheres and the cerebellum, is a series of ganglia, which have been called "sensory ganglia:" these are the corpora striata and the thalami optici; and the olfactive, optic, and auditory ganglia, which do not interest us at present. Though these have commonly been considered as mere appendages to the hemispheres, Carpenter maintains that they have an independent character, from the large quantity of vesicular matter they contain, and have special functions assigned to them. As we descend in the animal scale, we find these ganglia increasing at the expense of the cerebral hemispheres; we also, in the same ratio, find a less and less display of intelligence and will, and a greater predominance of the motions arising from instinct, that is, without any adaptation of means to ends. As in animals, so in man; in proportion as the reasoning powers are deficient (for it cannot be doubted that animals have a kind of intellect comparable with the reason of man), the instinctive impulses become stronger. As in the lower animals, so in the human idiot, the instinctive impulses, situated in these sensory ganglia, are strong, for his preservation from danger, and the supply of his necessary wants; though in this respect inferior to the animal, the human idiot, from want of power over the nerves and muscles, cannot always supply even his simplest wants. In these sensory ganglia is the seat of the instincts of animals, and the corresponding emotional actions in man: *to* the *thalami optici* as the focus of *sensation*, and *from* the *corpora striata* as the focus of *motion*, go the nerves which communicate the sensations, and the nerves which excite the motions, of instinctive and emotional actions. These actions being generally the most marked in idiots, we should expect to find these ganglia well developed in this class—not necessarily enlarged; as, if they were of the natural size only, they would undoubtedly be more active in proportion to the deficiency of the cerebral hemispheres. Whether pathological anatomy has decided this point, we are unable to say.

Phrenology has always claimed a peculiar connexion between the cerebellum and the *genital* system, and has adduced the frequent, perhaps general, activity of these functions in idiots in support of this view. Dr. Carpenter states that the weight of testimony, from comparative anatomy, experimental physiology and pathology, is decided in regard to the connexion of the cerebellum with the regulation of the *motor* function; though he does not totally deny the opinion of Dr. Gall. He adds, "It would seem by no means improbable that the lobes are specially connected with the regulation and co-ordination of movements; whilst the vermiform processes, which are very large in many animals in which the former scarcely present themselves, are the parts connected with the sexual function."

There is in idiocy an apparent contradiction as far as the cerebellum is concerned, inasmuch as there are frequently in the idiot strong sexual propensities, with a great want of order and control in the voluntary movements, and *vice versâ*. To explain this would require a combination, to say the least, of the phrenological doctrine with the views of Carpenter and others—and perhaps the entire separation of the sexual functions from the cerebellum.

It has been already seen that the idiot of Suabia (six years old) resembled very nearly, in shape and proportion of the skull, the Aztec children; and the phenomena of idiocy have now been sufficiently detailed to enable us to say why and to what extent these children are idiots. The measurements of the Suabian head are 14, $8\frac{1}{4}$, and $8\frac{1}{4}$ inches; the Aztecs have heads as small as new-born children, viz., 12, $7\frac{1}{2}$, and $7\frac{1}{2}$ inches, considerably smaller than the Suabian head. These children are now known to be dwarf specimens of a Central American race of Indians, such as may occur in any race; though no dwarfs on record have equalled these in the smallness of their crania. The brain seems merely too small, without any great disproportion in any of its parts; though, as usual, there is a relative inferiority of the anterior lobes, which may partly be accounted for by external circumstances with them favouring the development and exercise of the animal functions more than the intellectual. It is almost a harmonious want of development (if the expression be allowable), which gives them more the appearance of men in miniature than of idiots, though from the dwarfed condition of their brains they are necessarily partial idiots. The prominence of their features, though considerable, is exaggerated by their retreating foreheads; their bodies and extremities are well formed; they have good command over their muscles, and are quite agile, being continually in motion, differing in this respect from the majority of idiots. They certainly articulate words, and make a variety of animal-like noises, expressive of their wants, of anger, of joy, of surprise, and of other feelings, which imply considerable intelligence. They understand speech in others to a certain extent, as they obey like a little child; so that, as regards speech, as much seems to depend on an abnormal condition of the vocal organs, or the nerves supplying them, as on any intellectual defect. The senses are acute, especially sight and hearing; they are very attentive and curious, eagerly examining every new object. They in part feed themselves, and can chew solid food; they are decent in their habits, affectionate towards each other and to strangers; and they manifest desires and a degree of knowledge which place them high in the class of idiots, if not quite above them. The fact that the boy drivels, so characteristic of idiocy as to have become a byword, loses some of its significance when it is known that he is undergoing the process of his second dentition. Though they are dwarfs and idiots, yet they cannot be placed in the lowest classes; they exhibit such evident signs of intelligence, and are wanting in so many of the usual symptoms of idiocy, that we have little doubt that a judicious system of education would enable them to take a much higher rank among human beings than they now occupy.

Idiocy is, then, the inevitable result of a brain under certain dimensions. There are various lesions mentioned by authors as found in idiotic brains; among others, the small number and flatness of the convolutions of the cerebrum and cerebellum generally. Solly quotes from Breschet the case of a girl, fifteen years of age, in a complete state of idiocy, in whom the two anterior lobes of the brain were wanting; at the bottom of and behind the membranous pouch which replaced them, the corpora striata were seen exposed. In some cases, the brain seems hypertrophied; in others atrophied, with narrowness of the ventricles, so much insisted on by Esquirol; the convolutions may be found hardened, irregular, and discoloured, which Rostan thinks the result of softening followed by absorption: these lesions sometimes reach the optic thalami, the corpora striata, and the corpus callosum, and must have depended on arrest of development or intra-uterine disease of the brain, as they would soon have proved fatal if arising after birth.

Spurzheim says, "The brain of an idiot never resembles that of a sane person. Its form or texture is different." Even when the skull is well formed, as it is in many idiots from birth, the brain may be very small, and the interval be made up by a thickening of the bones. He mentions the skull of an idiot boy, which was three-fourths of an inch thick. The atrophy of the brain is usually accompanied by the atrophy of the extremities. An idiot examined by Esquirol presented the following symptoms, mentioned in Spurzheim's work on Insanity (pp. 243-4): The limbs of the right side were greatly atrophied, shorter than the left, and incapable of movement; the limbs of the left side were natural, and capable of voluntary motions; the head was small, but not deformed. On opening it, nearly all the gray cortical substance on both hemispheres was found wanting; instead of convolutions, there were only small irregular granulations; in regard to the white substance, that of the right hemisphere was natural, but in the left it was almost entirely wanting, being occupied by a sac of transparent fluid. This case has an interesting physiological bearing, as showing that the gray substance is not essential to voluntary motion, but is, as Sir C. Bell supposed, the seat of the intellect.

The pathological anatomy of "cretinism," by which is here understood any idiotic condition from causes subsequent to birth, must of course be very various. Only the most common and evident will be mentioned, and such as are suggested by those casts of hydrocephalic idiot heads in which the distortion reminds one of the Natchez Indian heads. The dimensions are very great, as will be seen from the following table:—

TABLE III.—*Hydrocephalic Heads*

| | 1st Measure. | 2nd Measure. | 3rd Measure. | Where found. | No. |
|---|---|---|---|---|---|
| Pimault. | 21 inches. | 15½ inches. | 16 inches. | Med. College, No. 86 | 28 |
| Do. | 19 „ | 12 „ | 15 „ | „ „ „ 87 | 29 |
| | 24¾ „ | 14 „ | 15½ „ | „ „ „ 106 *a* | 30 |
| | 25½ „ | 17 „ | 18 „ | „ „ „ 107 | 31 |
| Thick skull | 25 „ | 15½ „ | 16½ „ | „ „ „ 345 | 32 |

By comparing these with an average skull (No. 19), we see the other extreme of size in idiot heads. We shall not here detail the symptoms of chronic hydrocephalus, nor its morbid appearances, but only allude to one condition of the brain connected with the thickness of the cranial bones usually met with after the absorption of the cerebral fluid. The thick skull (No. 32) was that of a woman, who, at the age of fifty, enjoyed the use of all her faculties; from this time, her skull gradually thickened from disease, and her faculties became impaired in the same degree, till she died, at the age of sixty. The average thickness is about one inch; it is thickest at the sides and posteriorly, where it is an inch and a quarter thick; its thinnest part is one-half an inch thick. In the plates recently published of the diseased bones of the Dupuytren Museum in Paris, another specimen, equally remarkable, is accurately represented.

In some cases, thickening of the skull is undoubtedly the result of increased action in the vessels of the head. Dr. Combe noticed it in cases where there had been unusual activity of certain faculties, with increased cerebral circulation; and this is still more common in actual insanity. He mentions (on Mental Derangement, p. 259) a case where the brain had diminished in size in proportion to the increased thickness of the skull, and where the frontal convolutions, corresponding to the thickest part of the frontal bone, were proportionally smaller than in the rest of the brain. He gives several cases, in all of which the cerebral vessels were gorged with blood. Thickness of the skull may also occur in other diseases of increased action, as, for instance, in erysipelas of the head (*op. cit.* p. 262), in which there was unusual thickness in the occipital region. In some cases of thickening, the diploë is perfect, the increase being in the two tables; but in others everything is confounded in one thickened mass, which appears to have been the case in our specimen; the bone is said sometimes to be of an ivory hardness.

As we know exostosis is the result of a limited periostitis, there is no improbability in supposing that this general hyperostosis is the result of a general inflammation of this membrane. It would be difficult to account for it satisfactorily, when it occurs on the inner table, on any other hypothesis.

Chronic hydrocephalus affects the bones of the head in two ways: either the bones are thinned and softened from imperfect ossification, or they are thickened. According to Andral, this thickness is not accompanied by any great increase in weight, the compact bone being replaced by a spongy texture. The cause of thickening is supposed by Andral to be this: the quantity of liquid having reached its maximum, if life be prolonged, it begins to be absorbed; as the liquid disappears, in order that there may be no interval between the brain and bone (ossification preventing the depression of the bone towards the brain) new osseous deposits must be made on the internal surface of the cranium, according as the brain assumes its natural dimensions; so that externally the head preserves the hydrocephalic size, while the cranial cavity has only its normal capacity. When the effusion separates the bones, leaving a membranous space between them—if the subject lives to be adult—these membranous spaces are filled by ossa Wormiana, as has been shown by Rudolphi and Breschet; these are chiefly found at the superior angle of the occipital bone and along the lambdoidal suture, where separation would very likely be greatest, and here also is generally found the greatest thickening. It is not meant to be understood that only the above cause is concerned in the production of these supernumerary bones; but this is only one of many, though a more frequent one than is usually admitted.

From whatever cause the thickening proceeds, the manifestations of the mind are more or less disordered. Out of 216 cases of insane persons, Gredin found 167 who had thickening of the skull—seven-ninths of the whole.

These hydrocephalic heads show various shapes. Pimault had a flattening from front to back, like the Natchez head; of her it is said that she had shown a great deal of pride.—No. 30 was a well-formed head, though enlarged in all its diameters.—No. 31, the largest of all, was a child two or three years old, with great prominence of frontal region and vertex. An examination of the brain shows an anterior arrest of development; and a great flatness, after the evacuation of the water.

It will be observed, that in secondary idiocy there is no arrest of development, but a disorganization and disease in a brain previously healthy. Next to hydrocephalus as a cause of idiocy may be ranked the cerebral lesions of "cretinism," in its restricted sense. Dr. Pellissier, of Geneva (in Dr. Güggenbuhl's first report of the Abendberg Institution, p. 49), considers a false hypertrophy of the brain as the most probable cause of cretinism. This organ may undergo a kind of vesicular extension, without an actual serous effusion, which diminishes and flattens the convolutions; in other words, hypertrophy with dilatation. In this stage of the affection, the cranium is atrophied by this dilatation, the sutures are separated, and the fontanelles are widened. When the dilatation ceases, the brain again subsides, and the bone in proportion becomes hypertrophied; he thus accounts for the thickening of the skull, which he says almost always exists in cretins of an advanced age. In this period of false cerebral development, the intellectual faculties are greater than in healthy children of the same age; this makes the subsequent decline the more marked and painful.

The cretin head, as will be seen by comparing the following table with the preceding, is larger than the average head of the same age; as would be supposed from the hypertrophy which the brain undergoes. The measurements of the first five skulls are taken from Dr. Güggenbuhl's report above quoted; the last is No. 405 in the Medical College Collection.

TABLE IV.—*Cretins.*

| | Age. | 1st Measure. | 2nd Measure. | 3rd Measure. | No. |
|---|---|---|---|---|---|
| Marie S................ | 2 years. | 16 inches. | 13¾ inches. | 10½ inches. | 33 |
| Claudine S. ........... | 2 ,, | 18 ,, | 14¼ ,, | 12¼ ,, | 34 |
| John F. ............... | 3 ,, | 20¼ ,, | 16 ,, | 9½ ,, | 35 |
| Eliz. Z. .............. | 3 ,, | 19⅛ ,, | 13¾ ,, | 12¾ ,, | 36 |
| Martin D. ............. | 5 ,, | 20¾ ,, | 14⅞ ,, | 10 ,, | 37 |
| Cretin of the Vallais .... | aged. | 20½ ,, | 13 ,, | 11½ ,, | 38 |

In the last skull, the size is about the average; the antero-posterior diameter is somewhat longer, while the third measurement is correspondingly less; the bones are heavy, and, as far as can be judged without section, thicker than usual, analogous to the thickening of rachitic bones. On the sides of the occipital bone there is considerable prominence, with a depression on the median line; perhaps to be explained by the cerebral hypertrophy (of Dr. Pellissier) expanding the bones at the points of least resistance on the sides, the middle being supported and strengthened by the internal ridge for the sinuses and falx.

There is no need to more than mention the other forms of secondary idiocy, from various cerebral diseases, recognisable after death; and those more insidious forms from mere nervous exhaustion depending on severe study, venereal and other excesses, which may or may not elude our post-mortem search. These have been recognised as forms of dementia. One of the most common is believed to be from the premature tasking of the infant mind by our forcing-system of education, which, if it do not end fatally by cerebral disease, is liable to be followed by diminution of the intellectual powers, and even by hopeless idiocy.

The treatment of the various forms of idiocy and cretinism may be summed up in a few words. In an adult, who has been an idiot from birth, there is but slight hope of any great amelioration, as far as the brain is concerned, though the general health may be improved. All authors agree that *physical* treatment is most to be depended on, viz., pure air, gymnastic exercises, proper diet, and cleanliness. Medicines, except to correct ordinary symptoms, have not been attended with success. As a general stimulant to the nervous system, electricity and electro-magnetism would seem peculiarly applicable. Moral means are also of great value, as gentleness, kindness, and affectionate treatment.

As the animal instincts are here developed at the expense of the intellectual and moral sentiments, it becomes an object, if possible, to restore the equilibrium between these; if the higher feelings can be called into play, their animal nature will be proportionally lessened, as it were, by a kind of cerebral revulsion. The success of teachers has been found to be proportioned to their tact in interesting and fixing attention, that the rudiments of knowledge may be communicated; if *one* step be made in the right direction, it is comparatively easy to keep them in the path. Says Dr. Howe (page 54 of his Report), in the idiot, and in every one, "that which is, by nature, a little the strongest, becomes, *by exercise of its functions,* and by *neglect of exercise* of the functions of other parts, *very much* the *strongest,* until it utterly prostrates and masters them."

Dr. Combe (*op. cit.* p. 224) remarks, that the excitement of fever may restore the idiotic to reason. When the idiocy arises from cerebral inaction or weakness, the febrile paroxysm raises the activity of the brain to the height requisite for a vigorous exercise of its functions; when the paroxysm is over, the mental phenomena return to their former level. How far an artificially-produced febrile paroxysm, as by the agency of cold water, may be of advantage, would seem worthy of trial; the much-abused "vis medicatrix naturæ" might thus be stimulated in a natural and efficient manner. That disease, artificial or natural, may be of advantage in the various forms of *dementia,* may be conceived from the following analogous facts mentioned by Dr. C. H. Stedman, in the last Report of the Boston Lunatic Hospital, (p. 18.)

"One patient, an Indian, in good bodily health, afflicted with chronic mania, and who had been insane for three years, was seized with the severest form of dysentery which has ever come under my observation.

While in the height of the malady, his mental operations began to undergo a change. After which, his mental and bodily convalescence went on together, and resulted in the perfect restoration of the entire man. Another, a man who had been insane over twenty years, and quite a difficult one to manage, owing to his strong mischievous propensities, was attacked with the same affection, and remained dangerously ill for some weeks. He recovered from dysentery, and now no patient in the house is more quiet and controllable. Indeed, to many he would appear mentally sound."

If we examine the chemical constitution of the brain, we shall find a difference between the idiot and the normal condition, which it may be well to mention. From the researches of M. Couerbe, it appears that the proportion of phosphorus is much less in the idiot than in the normal brain. According to Carpenter, the contents of the nerve-cells and tubes are chiefly phosphorized fats; and he regards them as the active agents in the operations of the nervous system. The amount of phosphorus is greatest at the period of greatest mental vigour; in idiocy, the proportion is one-half less. This may indicate the internal exhibition of phosphorus in idiocy; it has long been known as an excellent general stimulant of the nervous system.

In Dr. Güggenbuhl's report, there are detailed several cases of great improvement in cretins from the treatment followed at the Institution on the Abendberg; this treatment is purely physical. In No. 33, after a residence of two and a half years, there was an increase in the first measurement of $2\frac{5}{8}$ inches; with a corresponding improvement in the intellectual faculties. For further details of a most satisfactory nature, this Report may be consulted with advantage.

Any interested in the improvement of idiots will be greatly astonished as well as gratified by a visit to the school at S. Boston, under the care of Mr. Richards. What volumes could not convince us of before the actual experiment, they will there acknowledge, viz., that kind treatment, perseverance, proper food, exercise, sports, and a judicious mental discipline, will do much to improve the condition of the hitherto abandoned class of idiots.

It has been seen that idiocy and partial talent may exist together, where, with a generally defective brain, certain portions are well developed; and in such cases, where the size and form of the head are changed, accurate measurements may be of great importance in a legal point of view. The law of Dr. Gall, then, may be repeated, in conclusion, viz., that when the first and second measurements of the head (as above defined) are below 17 and 11 inches, there is always greater or less stupidity; that when the first is from 11 to 13 inches, and the second 8 or 9, the condition can be but little above idiocy.

